# Neurogranin negatively regulates gene expression and proinflammatory mediator release in allergen-activated mast cells

**DOI:** 10.1093/immhor/vlag026

**Published:** 2026-05-28

**Authors:** Katie D Hunter, Robert W E Crozier, Ryan W Baranowski, Val A Fajardo, Adam J MacNeil

**Affiliations:** Department of Health Sciences, Faculty of Applied Health Sciences, Cairns Family Health and Bioscience Research Complex, Brock University, Niagara Region, ON, Canada; Department of Health Sciences, Faculty of Applied Health Sciences, Cairns Family Health and Bioscience Research Complex, Brock University, Niagara Region, ON, Canada; Department of Kinesiology, Faculty of Applied Health Sciences, Cairns Family Health and Bioscience Research Complex, Brock University, Niagara Region, ON, Canada; Department of Kinesiology, Faculty of Applied Health Sciences, Cairns Family Health and Bioscience Research Complex, Brock University, Niagara Region, ON, Canada; Department of Health Sciences, Faculty of Applied Health Sciences, Cairns Family Health and Bioscience Research Complex, Brock University, Niagara Region, ON, Canada

**Keywords:** allergy, chemokines, cytokines, inflammation, mast cells

## Abstract

Mast cells are critical players in the maladaptive immune responses underlying biphasic reactions in allergic inflammation. Neurogranin (Ng) is an IQ domain–containing protein that sequesters Calmodulin under low [Ca^2+^] and negatively regulates calmodulin-mediated signaling such as calcineurin activation, in addition to downstream inflammatory responses. Previously described as brain specific, Ng has recently been identified in the spleen, bone marrow, and B lymphocytes, highlighting the need to investigate its negative regulatory role in other proinflammatory contexts. Here, we sought to determine the role of Ng in allergen-activated mast cells, to better understand the negative regulatory mechanisms that can potentially be exploited to help alleviate the severity of allergic inflammation. Using bone marrow–derived mast cells from wild-type (*Nrgn*^+/+^) and heterozygous (*Nrgn*^+/−^) mice, we identified that Ng is present in mast cells. It was determined that although Ng did not influence the development of mature mast cells or the extent of early-phase inflammation, a reduction in Ng significantly increased gene expression of *IL6* and *IL13*, which was coupled with an increase in release of IL-6, IL-13, TNF, CCL1, CCL2, and CCL3. Together, this is the first study to identify Ng in mast cells, in addition to positioning its role as a negative regulator of mast cell responses following allergen activation. These data highlight the need for future research to further elucidate the role of Ng in IgE-mediated mast cell activation to better understand the regulatory mechanisms of these inflammatory cells in mast cell–driven normal and pathological contexts.

## Introduction

Widely distributed throughout vascularized tissues at the host-environment interface, mast cells are multifunctional immune sentinels largely responsible for the pathogenesis of allergic hypersensitivity reactions.^1^ Allergic inflammation is the result of a maladaptive immune response against an innocuous environmental antigen. Allergen-specific IgE antibodies strongly bind FcεRI receptors constitutively expressed on the surface of mast cells, sensitizing the cells for rapid activation upon subsequent re-exposures.^1^^,^^2^ Aggregation of FcεRI-IgE complexes through the recognition and binding of bi- or multivalent antigens in the presence of local stem cell factor (SCF) and initiates a series of biochemical events culminating in a time-dependent biphasic response.^3^^,^^4^ The immediate-phase (early) response is characterized by the release of preformed, granule-associated proinflammatory mediators that increase vascular permeability, smooth muscle contraction, and mucus secretion.^5^ The immediate response is followed hours later by a delayed, late-phase response, characterized by marked infiltrations of eosinophils, neutrophils, and lymphocytes, in addition to sustained local inflammation as a result of the continual production and release of proinflammatory mediators by allergen-activated mast cells.^5^^,^^6^

Upon FcεRI-IgE complex cross-linking with the cognate allergen, a positive signal is transmitted via immunoreceptor tyrosine-based activation motifs (ITAMs), rapidly inducing potent mast cell activation.^7^^,^^8^ Downstream signaling events lead to the production of inositol triphosphate (IP_3_) and diacylglycerol, critical secondary messengers in intracellular calcium (Ca^2+^) mobilization.^8^^,^^9^ Ca^2+^ signaling in response to allergen-mediated mast cell activation plays a pivotal role in degranulation, proinflammatory cytokine/chemokine production, and generation of eicosanoids.^10^ Proinflammatory gene expression is largely dependent on nuclear factor of activated T cells (NFAT) and nuclear factor κB (NFκB), transcription factors regulated by the Ca^2+^/calmodulin (CaM)-dependent, serine phosphatase calcineurin (CaN).^11^^,^^12^ Under resting conditions, these transcription factors are sequestered in the cytoplasm; NFAT in its phosphorylated, inactive state and NFκB bound to its inhibitor, IκB.^11^ Nuclear translocation of NFAT is dependent on dephosphorylation by CaN, mediated by the formation of NFAT-CaN complexes. CaN has also been experimentally shown to enhance activation of NFκB; however, the exact mechanisms remain incompletely defined.^13^^,^^14^ Immunosuppressive agents that inhibit the protein phosphatase activity of CaN have demonstrated reduced formation of the protein complex composed of Carma1, Bcl10, and Malt1 (CBM complex), an essential step in NFκB activation.^14–16^ Additionally, inhibition of CaN activity has been demonstrated to reduce IgE-mediated mast cell release of histamine and preformed proinflammatory mediators and shown to effectively treat allergic inflammation.^17^^,^^18^

CaM is a Ca^2+^ sensing protein that under conditions of elevated intracellular Ca^2+^ concentrations binds up to 4 Ca^2+^ ions, inducing a conformational alteration that allows CaM to regulate the activity of various proteins, enzymes, and ion channels.^10^^,^^19–21^ CaN is among the targets to which Ca^2+^-saturated CaM will bind and activate by displacing the autoinhibitory domain bound to the catalytic domain that inhibits its phosphatase activity.^19^^,^^20^ Activation of the phosphatase activity of CaN is thus dependent on the cellular availability of CaM and, furthermore, its ability to bind Ca^2+^ ions.^19^^,^^20^

Neurogranin (Ng) is an IQ domain–containing protein that binds and sequesters CaM under a low Ca^2+^ microenvironment, negatively regulating CaM-dependent signaling by limiting the cellular availability of CaM and decreasing its affinity for Ca^2+^.^22^^,^^23^ When intracellular Ca^2+^ levels increase, Ng dissociates from CaM as a result of weakened binding affinity for Ca^2+^-bound CaM and is subject to protein kinase C (PKC)–mediated phosphorylation of a serine residue within the IQ motif, further preventing its binding to CaM.^23–25^ In neuronal cells, Ng has been shown to suppress the phosphatase activity of CaN by limiting the cellular availability of Ca^2+^-saturated CaM.^24^ Despite previous investigations suggesting that Ng expression is restricted to brain tissue and neuronal cells, Ng expression has been discovered in endothelial cells,^26^ the thymus,^27^ spleen and bone marrow,^25^ and moderate levels in B lymphocytes.^28^

Calcium signaling is a highly conserved mechanism of signal transduction that is essential for CaN activation, a critical step in cytokine secretion following mast cell activation.^10–12^ Inhibitory signals that quench the mast cell response and limit undesirable inflammation following mast cell activation remain incompletely defined.^29^ Due to the presence Ng in immune cells and various tissues and its role in regulating CaN-dependent transcription factor activation, this study sought to investigate the presence and role of Ng in inhibitory signaling of IgE and SCF-mediated mast cell activation as a novel regulator of CaN activation.

## Methods

### Mice

Male and female C57BL/6J mice, Nrgn^+/+^ (wild-type [WT]) and heterozygous *Nrgn^tm1Kph^/Nrgn^+^* mice from the Mutant Mouse Resource and Research Centre (stock#043288-MU) were housed and maintained with standard diet and living conditions in the Brock University Comparative Biosciences Facility. All protocols were approved by the Animal Care Committee at Brock University in accordance with the guidelines of the Canadian Council on Animal Care.

### Genotyping

Endpoint polymerase chain reactions (PCRs) were performed to determine the genotype of the pups. Separate PCR reactions were performed for the *Nrgn* gene and *LacZ* gene, inserted in place of *Nrgn*. Following the PCR reaction, the amplified products were separated on a 1.3% agarose gel (Fisher Bioreagents; #BP160-100) and labeled with SYBRsafe DNA gel stain. Following separation for 45 min at 80 V, the gel was imaged using a Bio-Rad ChemiDoc Imaging System. Amplification of a 500 bp DNA product indicated the presence of the *LacZ* gene, and amplification of a 183 bp DNA product indicated the presence of the *Nrgn* gene. Primers used for PCR included *Nrgn For 5′-AGAGAGGCTGGTTCTGCAAG-3′ Rev 5′-CCAGAACACGCTGAGTTCAA-3′ and LacZ For 5′-AGAGAGGCTGGTTCTGCAAG-3′ Rev 5′-GACAGTATCGGCCTCAGGAA-3′.*

### Bone marrow–derived mast cell cultures

Bone marrow was isolated from the femur and tibia of C57BL/6 mice, Nrgn^+/+^ (WT), and heterozygous *Nrgn^tm1Kph^/Nrgn^+^* knockout mice. Hematopoietic stem cells were differentiated under the direction of IL-3 (10% WEHI-3B; ATCC) conditioned media^30^ consisting of RPMI 1640 (Gibco; 11875-093) supplemented with 10% Serum Plus II (Sigma-Aldrich; 14009C) and 1% penicillin/streptomycin (Gibco; 15140-122), 200 nm PGE_2_ (Sigma-Aldrich; P5640), and 50 nM 2-mercaptoethanol (Sigma-Aldrich; M7522) and maintained at 37 °C with 5% carbon dioxide (CO_2_). Following 5 wk in culture, mast cell maturity was assessed via flow cytometry.

### Mast cell sensitization and activation

Bone marrow–derived mast cells (BMMCs) were sensitized overnight with anti-TNP IgE supernatant (harvested from TIB-141 cells; ATCC). The following day, BMMCs were centrifuged at 340 *g* for 10 min at 4 °C, washed twice with RPMI 1640 to remove unbound IgE, and resuspended in RPMI 1640 supplemented with 10% Serum Plus II and 1% penicillin/streptomycin. Cells were stimulated with 100 ng/mL trinitrophenol bovine serum albumin (TNP-BSA) (Biosearch Technologies; T-5050) in combination with 100 ng/mL SCF (PeproTech; 250-03) and incubated at 37 °C, 5% CO_2_ for various time points mimicking the phases of an allergic reaction.

### Flow cytometry

BMMCs (0.5 × 10^6^ per staining condition) were collected and washed with 6 mL of immunofluorescence buffer (phosphate-buffered saline with 0.2% NaN_3,_ and 1% BSA). Cells were resuspended at a density of 0.5 × 10^6^ cells/mL and stained with fluorochrome-conjugated antibodies as per manufacturer guidelines (Sony Biotechnology) on ice for 1 h in the dark. Following incubation, BMMCs were washed with 600 µL of immunofluorescence buffer and fixed in 400 µL of 1% formalin in phosphate-buffered saline. Cells were analyzed on a Sony SH800S cell sorter (Sony Biotechnology). PE-conjugated anti-mouse FcεRIα (#1271535), Armenian hamster isotype control (#2604535), and FITC-conjugated anti-mouse CD117 (c-kit) (#1129025) and rat IgG_2b_ (#2603025) isotype control were purchased from Sony Biotechnology.

### β-hexosaminidase degranulation assay

BMMCs were sensitized overnight as described previously, with the exception of cold Hanks’ Balanced Salt Solution buffer (Gibco; 14025-092) supplemented with 0.1% BSA used in place of RPMI 1640. Cells were resuspended at a density of 4 × 10^6^ cells/mL, aliquoted (100 µL/well) into a 96-well tissue culture plate (Starstedt; 83.1835) in duplicate and stimulated for 20 min at 37 °C, 5% CO_2_. Following stimulation, BMMCs were centrifuged in plates for 10 min at 1152  *g* (Beckman Coulter; B80285AC) to separate the supernatant and pellet, prior to pellet lysis with 200 µL of 1% NP-40 (Bio Basic Canada; NDB0385) in Hanks’ Balanced Salt Solution buffer. Following centrifugation, 50 µL of both supernatant and lysed pellet was transferred to a new 96-well plate and incubated for 2 h at 37 °C, 5% CO_2_ in 50 µL of 1 mM 4-nitrophenyl-N-acetyl-β-D-glucoaminide (Sigma-Aldrich; N9376). Reactions were neutralized with 200 µL of 1.0 M carbonate buffer and the plate was read on a spectrophotometer (BioTek Synergy HT-1) at 405 nm wavelength. To account for culture-to-culture variation in maximal secretory output, degranulation was expressed as relative to maximal degranulation per culture (represented as 100%).

### Quantitative polymerase chain reaction

RNA was isolated using an RNeasy Plus Kit (Qiagen; 74136) and quantified on a NanoVue nanodrop instrument (GE). Complementary DNA was generated with 450 ng RNA using RNA to cDNA EcoDry premix (Takara Bio Inc, #6399549) in a SimpliAmp Thermal Cycler (Applied Biosystems; 228001548). Quantitative polymerase chain reaction (qPCR) assays were performed on an ABI StepOnePlus Real-Time PCR System instrument (Applied Biosystems; #4376592) with KAPA SYBR FAST (Sigma-Aldrich; KM4103) Master Mix and amplification efficiency-optimized primers (IDT) (Table S1). Threshold cycle (Ct) values were analyzed using the Ct method, with *HPRT* expression used as a reference gene.

### Enzyme-linked immunosorbent assay

Enzyme-linked immunosorbent assays were conducted to measure cytokine concentrations in cell free supernatants from stimulated BMMCs. Enzyme-linked immunosorbent assays were performed using DuoSet kits (R&D Systems; TNF DY410, IL-6 DY406, IL-13 DY413, CCL1 DY845, CCL2 DY479, CCL3 DY450, and CCL9 DY463) according to the manufacturer’s instructions. Assays were performed on a Nunc MaxiSorp flat-bottom 96-well plate (Thermo Fisher Scientific; 44-2404-21) and developed with 100 µL TMB substrate (BD Biosciences; 555214). Optical density was measured using a spectrophotometer (Bio-Tek) at a 450 nm wavelength and cytokine concentrations were determined from the standard curve.

### Western blotting

BMMCs (5–10 × 10^6^/condition) were lysed with a buffer containing 8 M urea, 2 M thiourea, 4% (*w/v*) CHAPS, and 1× protease and phosphatase inhibitor cocktail. Purified lysates (30 µg protein) were loaded onto 15% acrylamide gels (Bio-Rad; 1610156) and electrophoresed for ∼50 min at 200 V. Proteins were transferred to polyvinylidene disulfide membranes via transblot turbo (Bio-Rad), subsequently blocked with 5% BSA, and probed with anti-Ng primary (MilliporeSigma; 07-425-I) and an anti-rabbit secondary antibody from Cell Signaling Technology (7074). Clarity max ECL substrate (Bio-Rad; 170-5062) was applied to membranes, images were visualized on a Bio-Rad ChemiDoc imager (12003153), and band intensity was quantified using the corresponding Bio-Rad Image Lab software (version 2.3.0.07)..

### Statistics

Results from all experiments were expressed as mean ± SEM and analyzed using a 2-tailed unpaired *t* test or 2-way analysis of variance depending on the statistical comparisons being made, and Šidák’s post hoc test was used when making multiple comparisons. Results were considered significant if *P* < 0.05 between *Nrgn*^+/−^ and *Nrgn*^+/+^ BMMC cultures.

## Results

### Ng is present in mast cells and dispensable in early-phase degranulation

Ng is a negative regulator of CaM that was previously reported to exist exclusively in the brain; however, it has recently been documented in primary and secondary lymphoid organs, sites of leukocyte production and lymphocyte maturation, respectively.^25^^,^^27^^,^^28^ Despite the critical nature of Ca^2+^/CaM signaling for inflammatory responses,^31–33^ the presence of Ng has yet to be investigated in immune cells other than B cells.^28^ Because the functional mast cell response is highly dependent on Ca^2+^/CaM signaling,^10^ this study sought to determine the presence of Ng in mast cells and characterize its role in the mast cell response to allergen.

**Figure 1:**
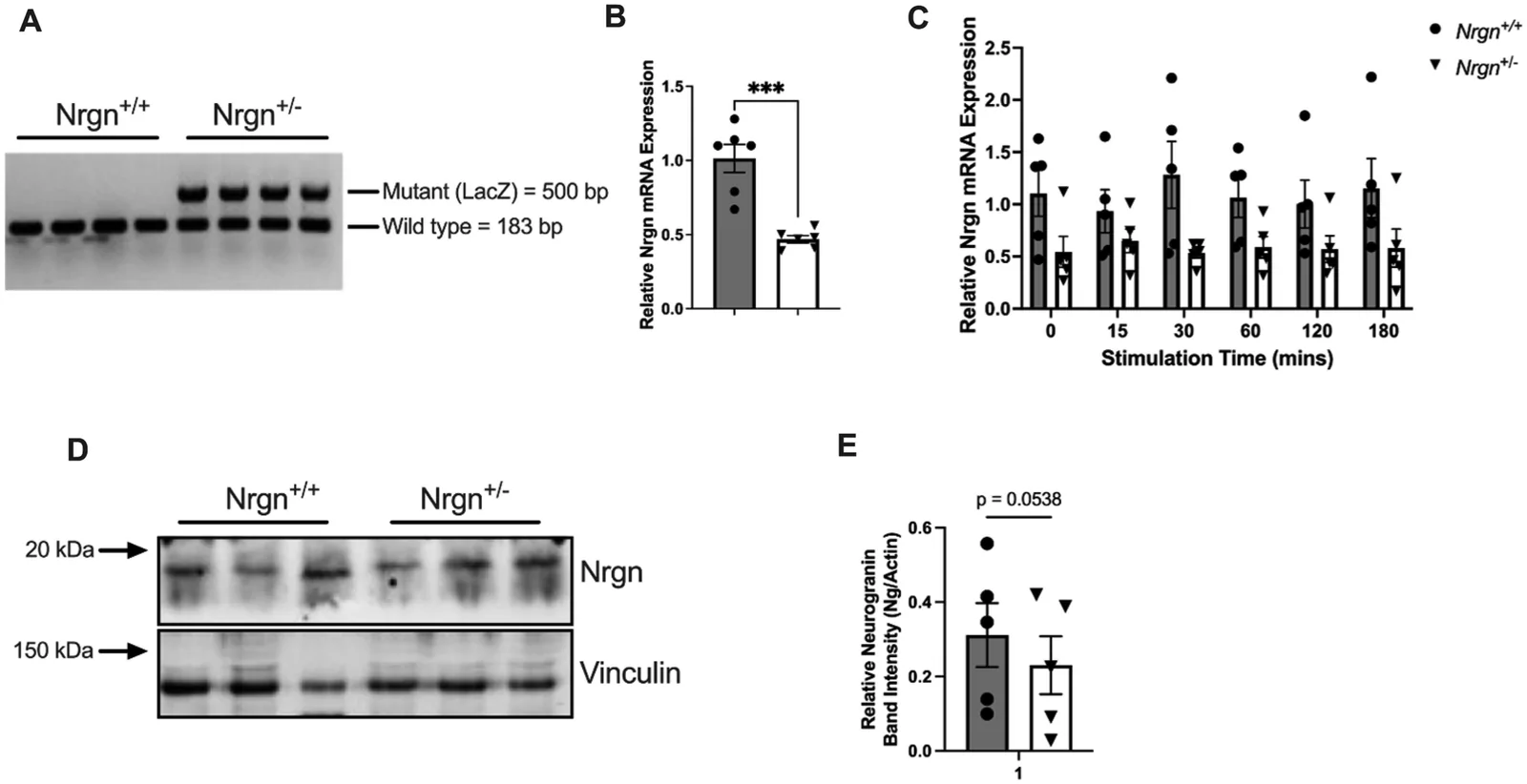
Nrgn is present in mouse mast cells differentiated from hematopoietic stem cells from Nrgn^+/+^ and Nrgn^+/−^ mice. Ear notches collected from male and female C57BL/6J *Nrgn^tm1Kph^/Nrgn^+^*, and *Nrgn^tm1Kph^*/*Nrgn^tm1Kph^* mice were subjected to endpoint PCR and the amplified products were visualized following DNA gel electrophoresis to determine the genotype. (A) The presence of a single band at 183 bp indicates a WT Nrgn^+/+^ genotype, while a band at 500 bp (LacZ) and 183 bp (Nrgn) indicates a Het Nrgn^+/−^ genotype. The DNA gel image is representative of 4 mice used from each group. Once the BMMCs were differentiated from isolated hematopoietic stem cells, qPCR was performed to confirm the presence of the Nrgn gene in both Nrgn^+/+^ and Nrgn^+/−^ mast cells (B) at baseline and (C) following allergen stimulation. Data are expressed as mean fold change ± SEM of 6 Nrgn^+/+^ and Nrgn^+/−^ cultures. (B) Unpaired *t* test and (C) 2-way analysis of variance were used to determine *Nrgn* gene changes between groups. ***P < 0.001 relative to Nrgn^+/+^ BMMCs. (D) Protein isolated from BMMCs was subjected to western blot analysis to confirm the presence of the Nrgn protein under resting conditions. (E) Relative Nrgn band intensity (Nrgn/Vinculin) was quantified to show differences in protein abundance between Nrgn^+/+^ and Nrgn^+/−^ genotypes. mRNA, messenger RNA.

To determine the presence of Ng and characterize its function, heterozygous Ng C57BL/6 mice (Nrgn^tm1Kph^/Nrgn^+^) were bred and the genotype of each mouse (ear notch) from the colony was subjected to endpoint PCR with primers specific to both the *Nrgn* gene as well as the *LacZ* gene, which was inserted in place of Ng within the mutant model (i.e. *Nrgn*^+/−^). The presence of 1 band at 183 bp (*Nrgn*) indicates a WT (*Nrgn*^+/+^) mouse, while the presence of a band at 500 bp (*LacZ*) and 183 bp (*Nrgn*) indicates that the mouse in heterozygous (*Nrgn*^+/−^) for the Ng gene (Fig. 1A). Once the appropriate genotypes were confirmed, BMMCs were generated from hematopoietic stem cells isolated from *Nrgn*^+/+^ and *Nrgn*^+/−^ mice. Following the 5-wk differentiation period, to determine the presence or reduction of Ng in mast cells, qPCR was performed to assess expression of the *Nrgn* gene in resting BMMCs (Fig. 1B). Here, we found that the *Nrgn* gene is present within mast cells and confirmed that BMMCs generated from *Nrgn*^+/−^ mice had a significant reduction in *Nrgn* messenger RNA relative to the *Nrgn*^+/+^ BMMCs (*P* = 0.0002) (Fig. 1B). These results were consistent with the gene expression profile of both genotypes following allergen stimulation. Here, we found that in both Nrgn^+/+^ and Nrgn^+/−^ BMMCs, gene expression of *Nrgn* did not change across the allergen stimulation time course, and a distinct difference was still observed between Nrgn^+/+^ and Nrgn^+/−^ genotypes (Fig. 1C). Following the discovery of the *Nrgn* gene expressed within mast cells, the presence of the Ng protein was assessed via Western blot to determine if protein levels followed the same trend at the gene level. Here it was found that under basal, unstimulated conditions, the Ng protein was observed within our mast cell model. (Fig. 1D). Coinciding with the observed reduction of *Nrgn* in Nrgn^+/−^ cells, Nrgn protein levels were ∼25% reduced in Nrgn^+/−^ cells compared with Nrgn^+/+^, though this did not quite reach statistical significance (Fig. 1D).

### Ng deficiency does not impact the phenotype of fully differentiated mast cells

**Figure 2:**
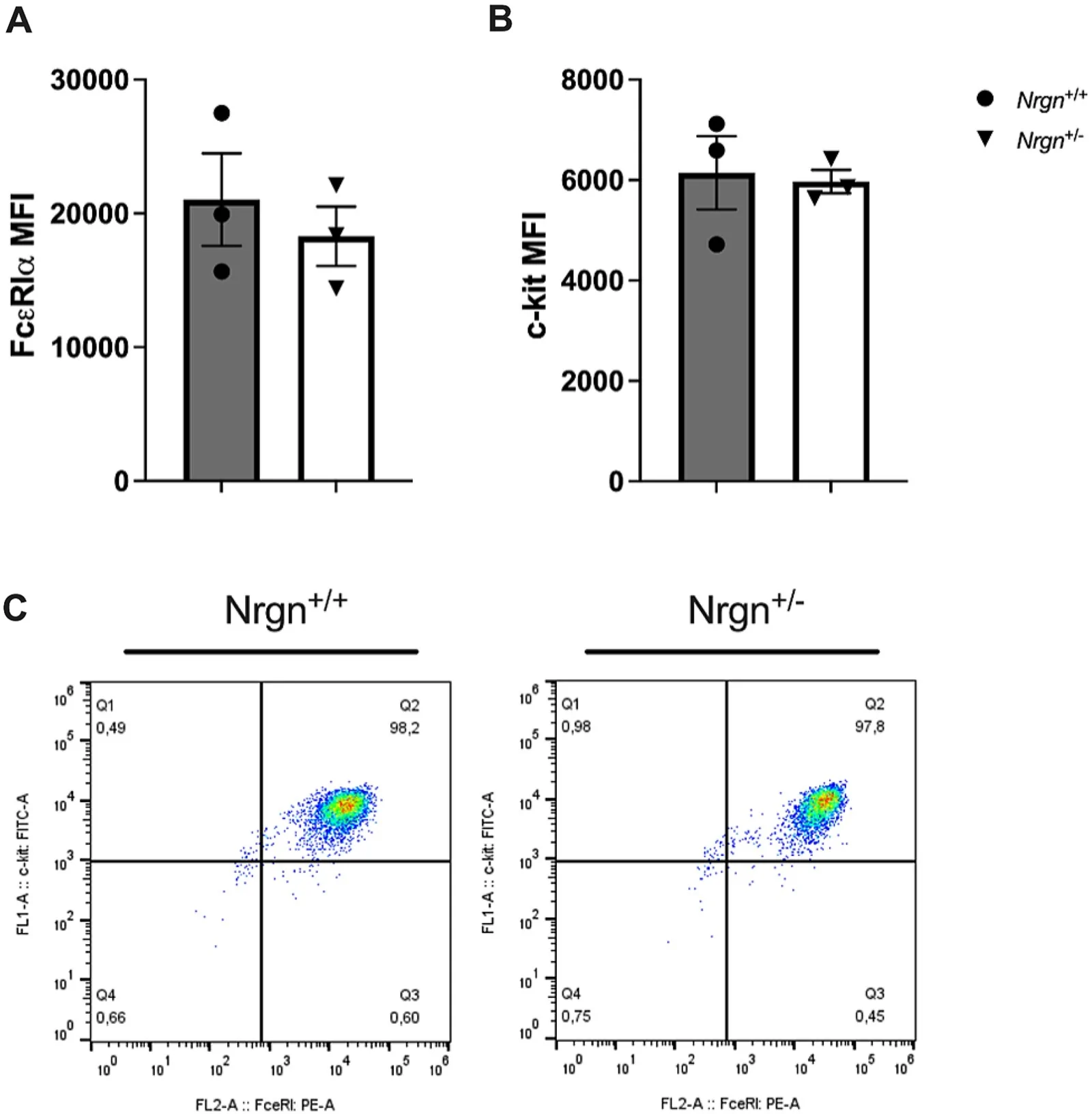
Neurogranin deficiency does not alter the abundance of phenotypically characterizing receptors on mature mast cells. (A) Representative image of coexpression of FcεRIα and c-kit in mature Nrgn^+/+^ and Nrgn^+/−^ mast cells. Mature mast cells were stained with anti-FcεRIα and c-kit antibodies for flow cytometric analysis to show the presence of (B) FcεRIα and (C) c-kit for each genotype. Data are expressed as mean fluorescence intensity from 3 individual Nrgn^+/+^ and Nrgn^+/−^ mast cell cultures. An unpaired Student *t* test was used to determine any differences between groups. MFI, mean fluorescence intensity.

Once it was determined that BMMCs express *Nrgn* at the genetic level and produce Nrgn protein, we wanted to determine if the reduction of Nrgn modulates the mast cell maturation process from hematopoietic stem cells. Mature mast cells are characterized by the constitutive expression of c-kit, the cognate receptor for SCF that regulates mast cell migration and survival, and FcεRI, the high-affinity IgE receptor.^34^ Therefore, we sought to determine if Ng deficiency affects the mature mast cell phenotype by assessing FcεRI or c-kit expression. BMMCs were independently fluorescently stained for FcεRIα (Fig. 2A) and c-kit (Fig. 2B) and subjected to flow cytometry. Following cytometric analysis, Ng deficiency was found to have no impact on either FcεRI or c-kit receptor surface levels, indicating that any downstream effects observed in Ng-deficient mast cells are not a result of altered receptor profiles in the receptor-mediated context of allergen stimulation.

**Figure 3:**
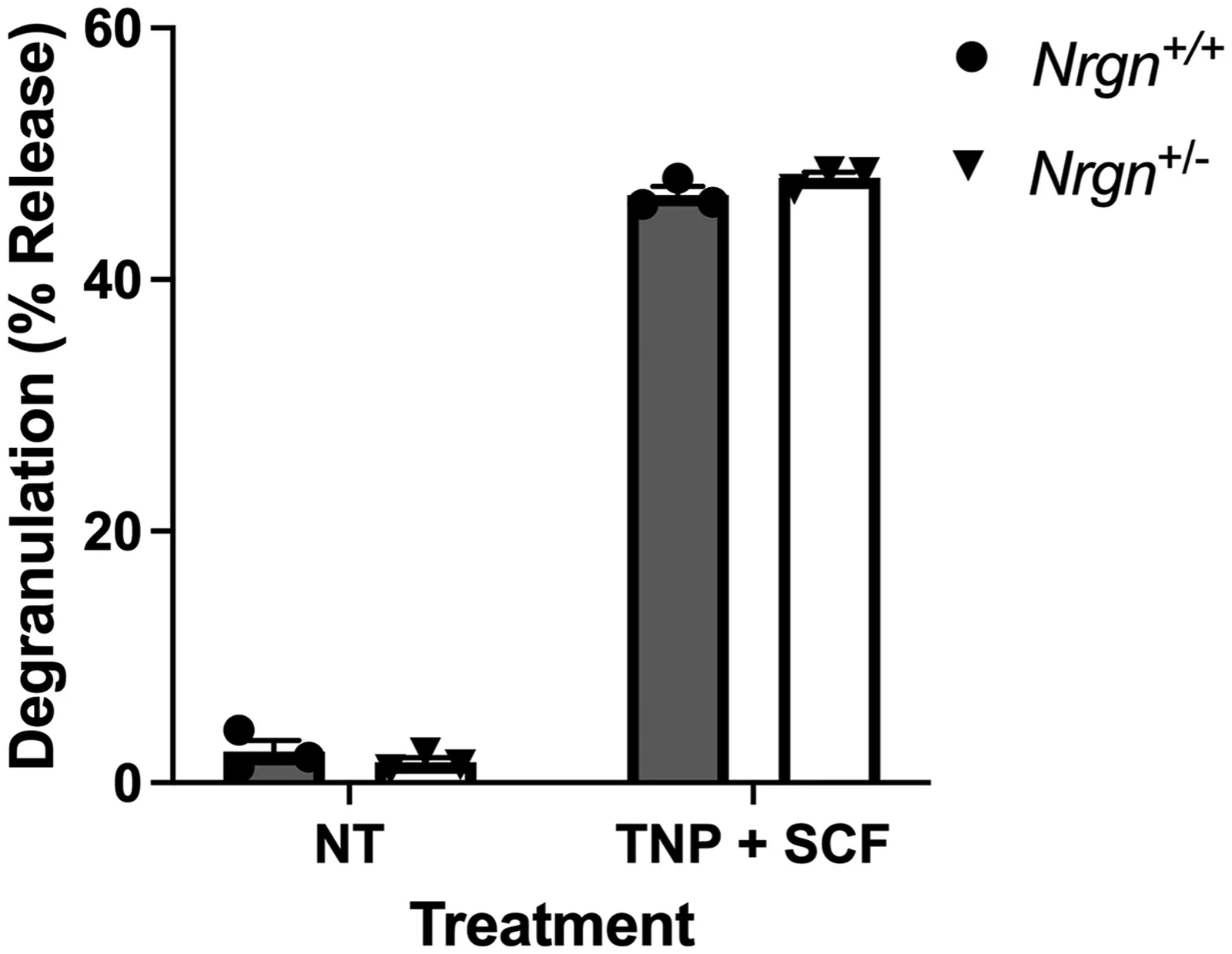
Early-phase degranulation remains unaltered in Nrgn-deficient mast cells. BMMCs sensitized overnight with IgE and stimulated with TNP-BSA + mouse SCF the following day were analyzed via β-hexosaminidase release assay to determine the extent of early-phase mast cell degranulation. β-Hexosaminidase release values are expressed as mean ± SEM of % release values [O.D. supernatant/(O.D. pellet + O.D. supernatant)*100] from 3 independent Nrgn^+/+^ and Nrgn^+/−^ mast cell cultures. An unpaired Student *t* test was used to determine if any differences between groups were present. O.D., optical density; NT, unstimulated BMMCs.

In addition to the highly characterized receptor “ome” of mature BMMCs, IgE-sensitized, mature mast cells are most notably recognized by their ability to immediately release a plethora of proinflammatory mediators upon allergen exposure. Following allergen-activated mast cell activation, there is an initial wave of Ca^2+^ mobilization due to a transient release of Ca^2+^ from endoplasmic reticulum stores,^10^^,^^35^ followed by a sustained influx and elevation of intracellular Ca^2+^ through store-operated calcium entry.^10^^,^^36^ Early-phase mast cell degranulation is a process that is highly dependent on the Ca^2+^ ions released from endoplasmic reticulum stores, which are essential for granule translocation and zippering.^10^^,^^37^^,^^38^ Because Ng is directly involved in the CaM/CaN pathway, we next sought to determine the role of Ng in the early-phase mast cell response. To characterize Ng deficiency in mast cell degranulation, release of the preformed lysosomal enzyme β-hexosaminidase was measured following TNP-specific IgE sensitization and subsequent stimulation with TNP-BSA and SCF for 20 min. Here, we found that Ng deficiency did not impact the amount of β-hexosaminidase released from BMMCs (Fig. 3), indicating that Ng does not required in early-phase mast cell degranulation.

### Ng differentially regulates allergen-induced proinflammatory gene expression in mast cells

**Figure 4:**
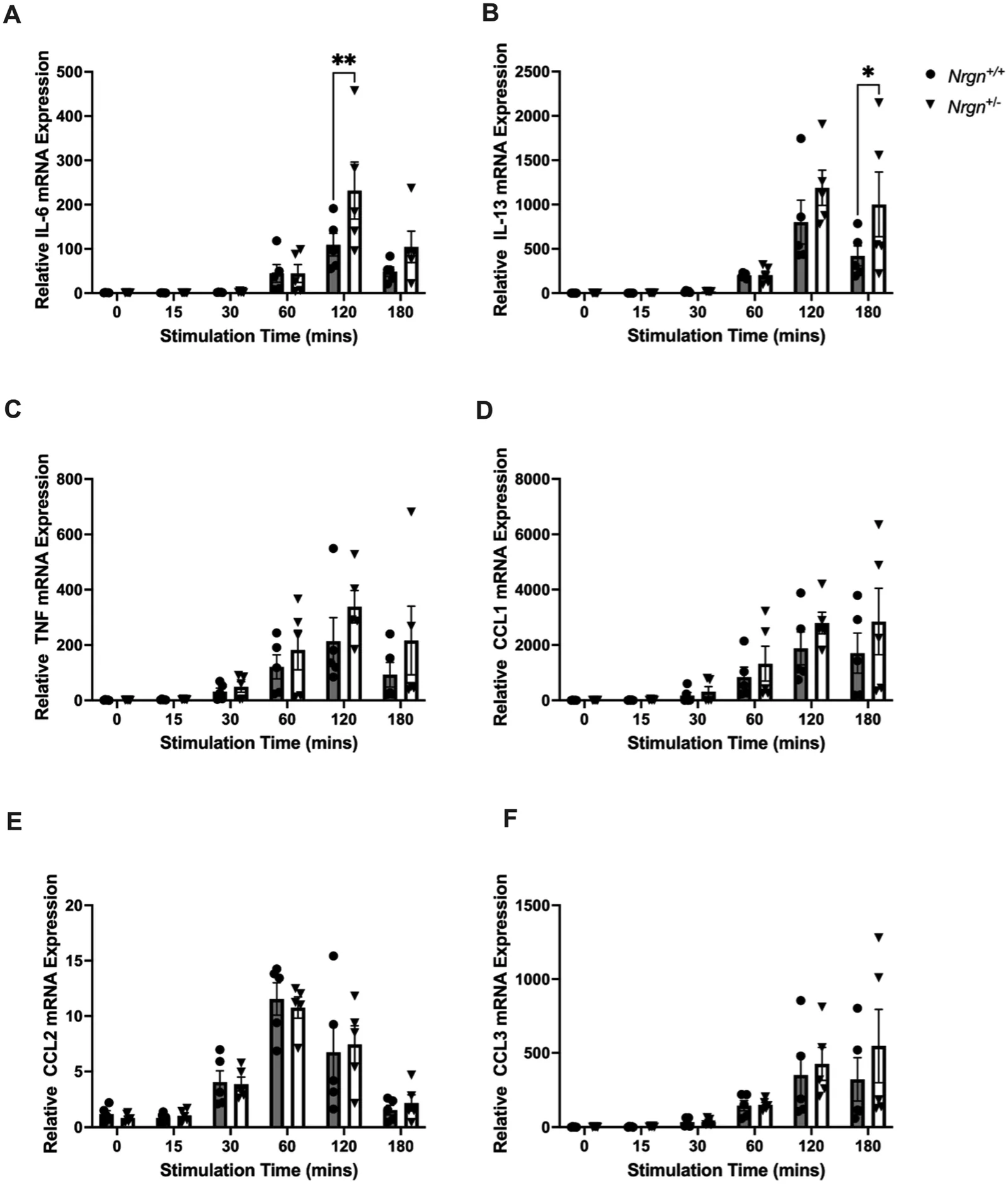
A reduction in Nrgn differentially regulates gene expression of allergen-induced proinflammatory mediators. Messenger RNA isolated from BMMCs stimulated with TNP-BSA + mSCF for 0, 15, 30, 60, 120, and 180 min was subjected to qPCR analysis. Here, gene expression changes of (A) *IL6*, (B) *IL13*, (C) *TNF*, (D) *CCL1*, (E) *CCL2*, and (F) *CCL3* were measured from 5 independent Nrgn^+/+^ and Nrgn^+/−^ mast cell cultures. Data expressed as mean fold change ± SEM. A 2-way analysis of variance and a Šidák’s multiple comparisons test were used to determine differences in gene expression between Nrgn cultures. *P < 0.05 and **P < 0.01 relative to Nrgn^+/+^ BMMCs.

Following the aggregation of FcεRI receptors through the binding of allergen, downstream signaling cascades are activated which ultimately leads to the transcription of proinflammatory genes.^39^ To characterize the role of Ng in the transcription of proinflammatory genes, qPCR was performed with RNA isolated from IgE-sensitized BMMCs stimulated with TNP-BSA and SCF for 0, 15, 30, 60, 120, and 180 min. The gene expression of *IL6* was found to be significantly increased (*P* = 0.005) at 120 min following stimulation in *Nrgn*^+/−^ BMMCs compared with *Nrgn*^+/+^ BMMCs (Fig. 4A). Additionally, increased gene expression of *IL13* (*P* = 0.04) was observed at 180 min in *Nrgn*^+/−^ BMMCs compared with *Nrgn*^+/+^ BMMCs following stimulation (Fig. 4B). Furthermore, there was a significant difference in the gene expression of *TNF* (*P* = 0.04) between genotypes (statistically significant column effect) and an observed biological increase in the gene expression of *CCL1* (*P* = 0.09) (Fig. 4C, D), with no observed difference in the gene expression of *CCL2* and *CCL3* (Fig. 4E, F) in *Nrgn*^+/−^ BMMCs compared with *Nrgn*^+/+^ BMMCs. Together, these findings indicate that Ng has a differential role in negatively regulating the transcription of proinflammatory genes in response to allergen.

### Ng deficiency augments allergen-induced cytokine and chemokine release

**Figure 5:**
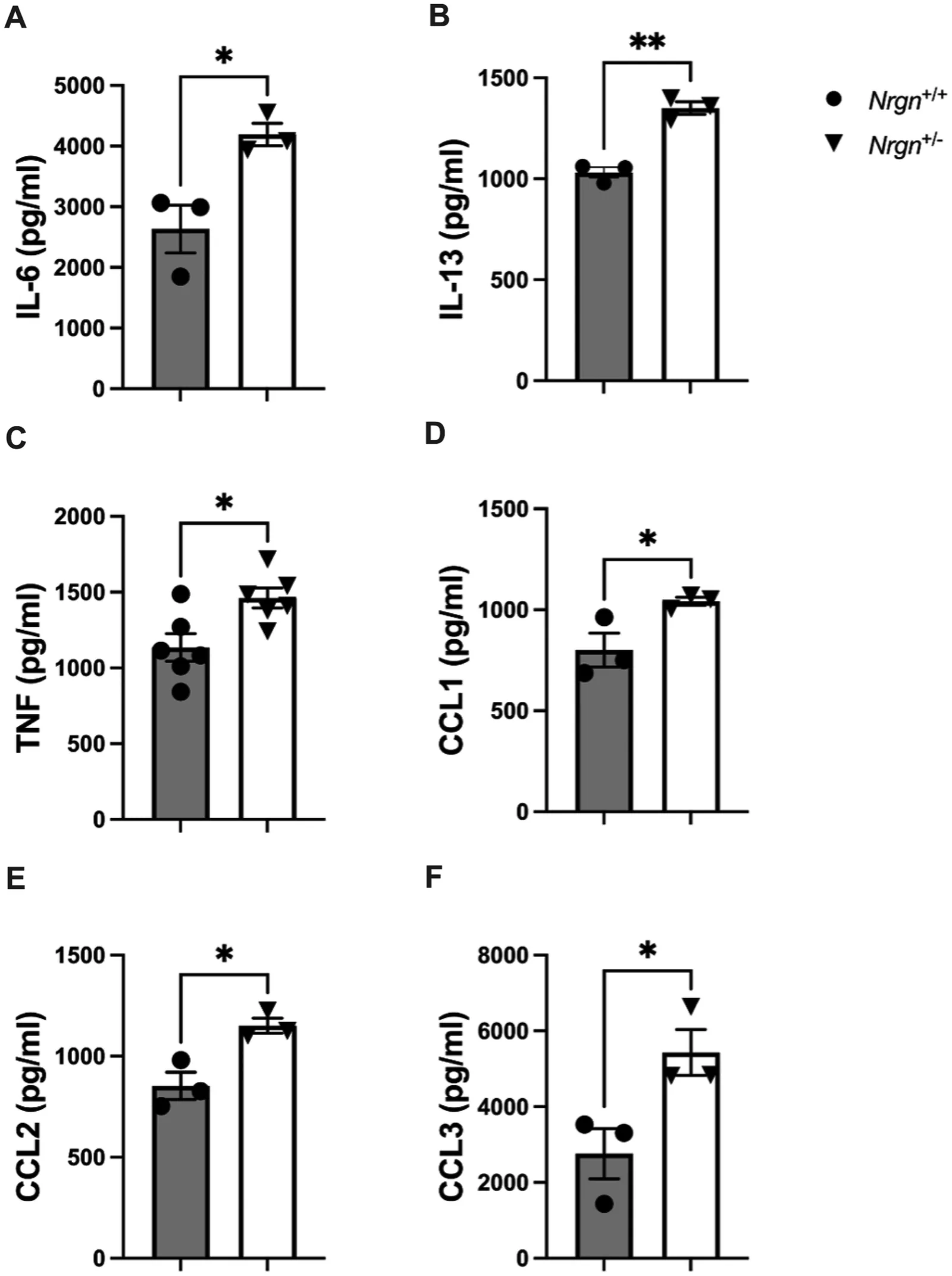
Proinflammatory mediator release is increased in Nrgn-deficient mast cells. Cell-free supernatants collected from TNP-BSA + mouse SCF–stimulated Nrgn^+/+^ and Nrgn^+/−^ BMMCs were analyzed via enzyme-linked immunosorbent assay to quantify protein release of (A) IL-6, (B) IL-13, (C) TNF, (D) CCL1, (E) CCL2, and (F) CCL3 following 6 h stimulation. Data are expressed as mean relative mediator release (mean percentage of control [WT]) of 3 to 6 independent Nrgn^+/+^ and Nrgn^+/−^ mast cell cultures. An unpaired *t* test was used to determine differences in mediator secretion from Nrgn^+/+^ and Nrgn^+/−^ mast cells. *P < 0.05 and **P < 0.01 relative to Nrgn^+/+^ BMMCs.

Building on our previous observation that Ng-deficient BMMCs exhibited elevated transcription of proinflammatory genes following allergen activation, we next assessed whether Ng deficiency also enhances proinflammatory cytokine and chemokine secretion. IgE-sensitized BMMCs were stimulated with TNP-BSA and SCF for 6 h, after which supernatant samples were collected for ELISA. Following allergen stimulation, Ng-deficient BMMCs released significantly higher levels of IL-6, IL-13, TNF, CCL1, CCL2, and CCL3 compared with WT *Nrgn*^+/+^ controls. Specifically, IL-6 secretion was approximately 1.6-fold higher in *Nrgn*^+/−^ BMMCs at 6 h poststimulation (*P* = 0.02), indicating a negative regulatory role for Ng in the secretion of IL-6 from mast cells (Fig. 5A). Similarly, IL-13 (*P* = 0.002) and TNF (*P* = 0.02) levels were significantly elevated in *Nrgn*^+/−^ BMMCs (*P* = 0.002 and *P* = 0.02, respectively) (Fig. 5B, C). These results were consistent with the release of chemokines CCL1 (*P* = 0.04), CCL2 (*P* = 0.02), and CCL3 (*P* = 0.04), all of which exhibited increased levels of secretion following allergen stimulation in *Nrgn*^+/−^ mast cells (Fig. 5E, F). Collectively, these findings demonstrate that reduced Ng expression enhances allergen-induced secretion of key proinflammatory mediators, underscoring its role as a negative regulator of mast cell–mediated inflammatory responses.

## Discussion

Ng is an IQ domain–containing protein that functions to bind and sequester CaM during periods of elevated Ca^2+^ concentrations, thus regulating Ca^2+^/CaM-dependent functional processes.^24^^,^^25^ Until recently, our understanding of the function of Ng was limited to research performed with brain samples as it was believed that Ng exclusively existed in the brain. Recent discoveries have shown that Ng exists in skeletal and cardiac muscle, endothelial cells, primary and secondary lymphoid organs, and B cells.^25^^,^^27^^,^^28^^,^^40^ Despite the presence of Ng in immune tissues, Ng expression has not been examined in immune cells other than B cells. Thus, given the importance of Ca^2+^ signaling in mast cell biology, this study first sought to investigate the presence of Ng in mast cells, and our resultant findings are the first to demonstrate significant Ng gene and protein expression during nonstimulated, basal mast cell conditions and define Ng as a novel negative regulator of mast cell inflammatory mechanisms driven by allergen.

CaM is an indispensable transducer of Ca^2+^ signals in mast cells that can bind up to 4 Ca^2+^ ions during periods of elevated cytosolic Ca^2+^ concentrations.^41^ When intracellular Ca^2+^ concentrations are elevated, Ca^2+^-CaM complexes have the ability to bind and activate many downstream targets. Among these targets is CaN, which upon activation through Ca^2+^-CaM binding transmits signals to the nucleus by mediating the nuclear translocation of critical proinflammatory transcription factors, NFAT and NFκB.^14^^,^^42^ Due to the indispensable nature of Ca^2+^/CaM/CaN signaling in inflammatory responses, in this study we investigated the role of Ng in the allergen-induced mast cell functional response using an Ng-deficient BMMC model.

Mast cells can be phenotypically characterized by the constitutive expression of the high-affinity IgE receptor FcεRI and the SCF receptor c-kit.^43^ Mast cell activation through allergen binding to IgE-FcεRI complexes in the presence of local SCF initiates a series of biochemical events that ultimately lead to a biphasic allergic inflammatory response.^44^ Our results demonstrated that Ng deficiency did not affect FcεRI or c-kit receptor expression, indicating that any functional effects observed with Ng deficient BMMCs were not the result of altered receptor expression. This is the first study to investigate the role of Ng in receptor expression.

Early-phase mast cell degranulation is a process that is highly dependent on Ca^2+^ ions, which are essential for granule zippering and the subsequent release of proinflammatory mediators.^10^^,^^37^^,^^38^ As measured through β-hexosaminidase release, an enzyme present within mast cell granules, Ng deficiency was found to have no effect on early phase mast cell degranulation. This is an interesting and unexpected finding as the CaM/CaN pathway has been demonstrated to play a role in mast cell degranulation.^45^^,^^46^ Previous research in RBL-2H3 cells, rat basophilic leukemia mast cells, showed that pretreatment with the CaM antagonist W-13 decreased β-hexosaminidase release following stimulation with ionomycin, suggesting that CaM plays a positive regulatory role in mast cell degranulation.^46^ Downstream, Ca^2+^/CaM complexes bind and activate additional signaling molecules such as CaN, Ca^2+^/CaM-dependent protein kinase, and myosin-light chain kinase.^47^ Further research in RBL-2H3 cells pretreated with cyclosporin A, KN-93, and ML-7—CaN, CaM-dependent protein kinase, and myosin-light chain kinase inhibitors respectively—each independently reduced β-hexosaminidase release when the cells were stimulated with ionomycin, suggesting a positive regulatory role for each of these major CaM targets in mast cell degranulation.^46^ Consistent with this research are findings in Rcan1-deficient BMMCs that show in the absence of Rcan1, a negative regulator of CaN, β-hexosaminidase release is enhanced following TNP-BSA stimulation, though Rcan1 expression is induced following stimulation versus constitutive for Nrgn, making the mechanism Rcan1 contributes through unclear.^45^ Thus, as Ng is known to bind and sequester CaM, preventing the activation of downstream Ca^2+^-CaM targets,^24^^,^^25^ Ng deficiency was hypothesized to enhance mast cell degranulation. The observed lack of effect of Ng deficiency on mast cell degranulation suggests potential compensatory mechanisms at play. Interestingly, however, there was an observed increase in *Rcan1* gene expression in Nrgn^+/−^ mast cells following allergen stimulation (Fig. S1). This is suggestive that other negative regulators of CaM/CaN signaling may be compensating the reduction in Nrgn to alleviate some of the proinflammatory responses observed in this study and that Nrgn may in fact negatively regulate induced Rcan1 expression itself.

During the late phase, Ca^2+^ signaling is also critical for the transcription of proinflammatory cytokines/chemokines following allergen stimulation.^39^ At the messenger RNA level, Ng deficiency was observed to increase IL-6 and IL-13 gene expression and additionally resulted in an increasing trend in TNF, CCL1, and CCL3, with no effect on CCL2. These findings indicate that Ng may negatively regulate CaN indirectly through CaM as they are consistent with those observed in Rcan1-deficient BMMCs, which found a significant increase in IL-6, IL-13, and TNF messenger RNA expression following TNP-BSA stimulation.^45^ CaN is responsible for directly dephosphorylating and activating NFAT, a major transcription factor that has been experimentally shown to regulate the transcription of IL-13, TNF, and IL-4.^48^^,^^49^ Furthermore, CaN has been demonstrated to promote the nuclear translocation of NFκB, another major transcription factor for proinflammatory genes that has been shown to regulate IL-6 and TNF.^50^ Therefore, the effects of Ng on proinflammatory gene expression are potentially due to a negative regulatory role on the activation of CaN and thus the activation and nuclear translocation of NFAT and NFκB, although future studies will have to confirm this mechanism in an allergen-activated mast cell model.

Ultimately, the proinflammatory cytokines and chemokines that are secreted following allergen activation are responsible for the signs and symptoms of the late-phase response.^44^ Similar to the gene expression changes, Ng was found to negatively regulate all key proinflammatory mediators investigated in this study. These findings are also in agreement with studies with Rcan1-deficient BMMCs that showed that in the absence of a CaN regulator, IL-6, IL-13, and TNF secretion is significantly enhanced.^45^^,^^51^ Furthermore, mouse liver–derived mast cells deficient in CaN subunit A stimulated with TNP-BSA show a decrease in TNF, IL-4, IL-6, and IL-13 secretion, further confirming the critical role of CaN in the secretion of proinflammatory cytokines.^52^ Together, these results further suggest that Ng is a negative regulator of proinflammatory cytokine and chemokine production and secretion and potentially functions through the indirect negative regulation of CaN activation.

Currently, various CaN inhibitors such as tacrolimus and pimecrolimus are being used as therapeutic options for the treatment of atopic dermatitis.^53^ Tacrolimus and pimecrolimus are both commercially available as topical ointments for the treatment of moderate-to-severe and mild-to-moderate atopic dermatitis, respectively. Studies have shown that tacrolimus decreases serum IgE, inhibits IgE-mediated histamine release from mast cells and basophils, suppresses T cell proliferation and production of cytokines, and inhibits bronchoconstriction in models of asthma.^54^ Similarly, pimecrolimus has been shown to inhibit both T cell and mast cell activation, as well as the activation of eosinophils.^55^ Therefore, the identification of Ng as a potential negative regulator of CaN activation in mast cells is a novel finding that provides crucial insight of clinical relevance into the molecular mechanisms of mast cell activation in response to allergen.

In summary, the presented data are the first to identify Ng as a protein present within mast cells and provide evidence that Ng negatively regulates the mast cell inflammatory response to allergen stimulation. Our findings demonstrated that Ng primarily regulates the late-phase mast cell response through regulation of the transcription of critical proinflammatory cytokines IL-6 and IL-13, along with the secretion of IL-6 and TNF. These findings warrant further investigation into the molecular mechanisms and role of Ng at an in vivo level.

## Supplementary Material

vlag026_Supplementary_Data

## Data Availability

All data are available from the corresponding author upon request.
