# Supplementary material for: Neurogranin negatively regulates gene expression and proinflammatory mediator release in allergen-activated mast cells
Source: Immunohorizons. 2026 May 28;10(5):vlag026. doi: 10.1093/immhor/vlag026 (PMC13218791; doi:10.1093/immhor/vlag026)
Supplement: vlag026_Supplementary_Data [file vlag026_supplementary_data.pdf]

Supplemental data

Table S1. List of qPCR primer sequences used. Mouse primers were designed using the NCBI Primer-BLAST function. Primer sequences were selected and custom DNA oligos were ordered from Integrated DNA Technologies at 25 nmole DNA oligo scale with standard formulation and standard desalting performed.

**Primer**	**Gene**	**Direction**	**Sequence (5' → 3')**	**NCBI Accession Number**
mHPRT_631F	HPRT	Forward	CTTGCTGGTGAAAAGGACCTCTCG	NM_013556.2
mHPRT_811R		Reverse	CGCTCATCTTAGGCTTTGTATTTGG	
mNrgn_179F	Nrgn	Forward	ACCCTCAACAACGGCAATGGA	NM_022029
mNrgn_292R		Reverse	ATTTTGGCTGCAGCGGCGT	
mTNF_319F	TNF	Forward	TGAACTTCGGGGTGATCGGTCC	NM_001278601
mTNF_436R		Reverse	TCCAGCTGCTCCTCCACTTGGT	
mIL6_439F	IL6	Forward	AGACAAAGCCAGAGTCCTTCAGAGA	NM_031168
mIL6_599R		Reverse	TGGTCTTGGTCCTTAGCCACTCC	
mIL13_101F	IL13	Forward	TGCTTGCCTTGGTGGTCTCG	NM_008355
mIL13_240R		Reverse	TCCATACCATGCTGCCGTTGC	
mCCL1_18F	CCL1	Forward	CCAGACATTCGGCGGTTGCT	NM_011329
mCCL1_150R		Reverse	CAAGCAGCAGCTATTGGAGACCGT	
mCCL2_72F	CCL2	Forward	CCTCCACCACCATGCAGGTCC	NM_0011333
mCCL2_189R		Reverse	CAGCAGGTGAGTGGGGCGTTA	
mCCL3_180F	CCL3	Forward	GGAGCTGACACCCCGACTGC	NM_011337

mCCL3_405R		Reverse	GGGTTCCTCGCTGCCTCCAA	

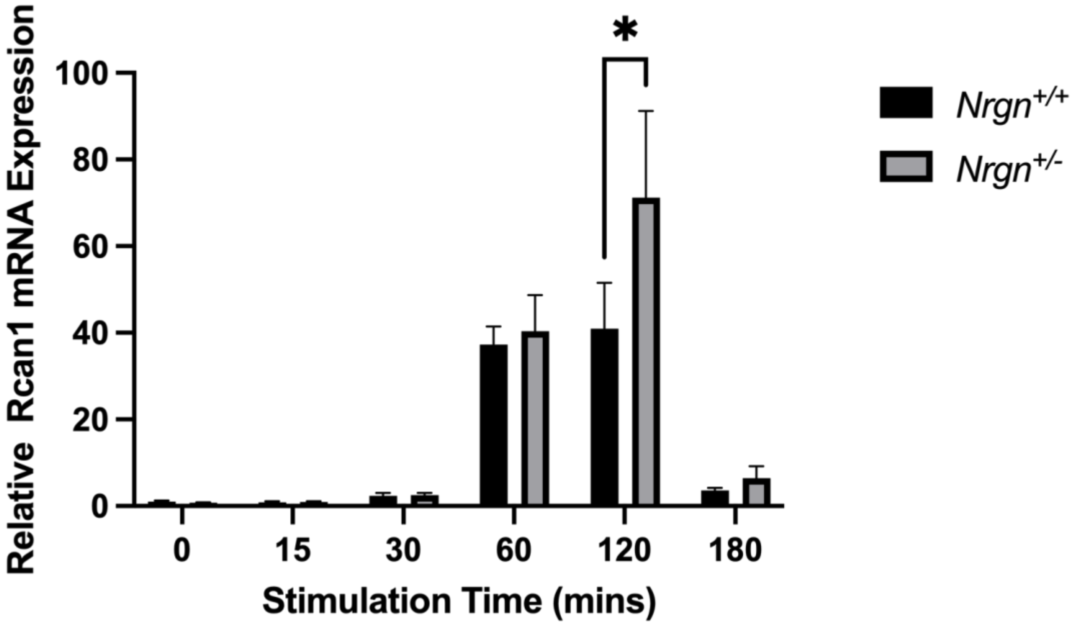

Figure S1. A reduction in Nrgn results in increase RCAN1 gene expression following allergen activation. mRNA isolated from BMMCs stimulated with TNP-BSA + mSCF for 0, 15, 30, 60, 120 and 180 mins was subjected to qPCR analysis to determine gene expression changes of *RCAN1*. Data expressed as mean fold change ±SEM of n=5 independent $Nrgn^{+/+}$ and $Nrgn^{+/-}$ mast cell cultures. A two-way ANOVA and a Šidák's multiple comparisons test were used to determine differences in gene expression between Nrgn cultures. * $p<0.05$ relative to $Nrgn^{+/+}$ BMMCs.
